# Mechanism of peptides from rice hydrolyzed proteins hindering starch digestion subjected to hydrothermal treatment

**DOI:** 10.1038/s41538-022-00153-3

**Published:** 2022-08-25

**Authors:** Xiaoxue Lu, Rongrong Ma, Jinling Zhan, Zhengyu Jin, Yaoqi Tian

**Affiliations:** 1 State Key Laboratory of Food Science and Technology, Jiangnan University, 1800 Lihu Road, Wuxi, 214122 China; 2 School of Food Science and Technology, Jiangnan University, 1800 Lihu Road, Wuxi, 214122 China; 3 National Engineering Research Center of Cereal Fermentation and Food Biomanufacturing, Jiangnan University, Wuxi, 214122 China

**Keywords:** Nutrition, Plant sciences

## Abstract

Clarifying the interactions between food components is critical in designing carbohydrate-based foods with low digestibility. To date, the hindering effect of starch-protein interactions on starch digestion has attracted extensive attention. In this study, rice proteins were further hydrolyzed, and rice peptides (RP) with different molecular weights were obtained by ultrafiltration. The effects and possible mechanisms of RP with different molecular weights on the structure, thermal properties, and in vitro digestibility of cooked rice starch were investigated. All peptides slowed the digestion of rice starch in a concentration-dependent manner. A concentration of 10% RP_>10_ decreased the rapidly digestible starch content from 68.02 to 45.90 g/100 g, and increased the resistant starch content from 17.54 to 36.54 g/100 g. The addition of RP improved the thermal stability of the starch and reduced the amount of leached amylose. Infrared analysis shows that strong hydrogen bonds formed between RP (especially RP_>10_) and starch during co-gelatinization. In addition, RP improved the compactness of aggregated structure and played an important role in hindering the enzymatic hydrolysis of starch. These results enrich the theory of starch-protein interactions and have important implications for the development of carbohydrate-based foods with low digestibility and protein functional foods.

## Introduction

Rice (*Oryza sativa* L.) is a staple food for half of the world’s population, and starch is the main carbohydrate in rice that produces blood glucose^1^. Starch-based foods are usually consumed after cooking, which rapidly and enzymatically breaks down in the body and causes a rapid rise in blood sugar in a short period^2^. Reducing the rate and extent of starch hydrolysis can help reduce the risk of diet-related diseases such as Type II diabetes, obesity, and cardiovascular disease^3^. Previous studies^4,5^ have shown that interactions between food components could affect the in vitro digestibility of starch.

In vitro and in vivo studies^6–9^ have shown those plant proteins or their hydrolysates could inhibit starch digestion, reduce glucose release and lower blood glucose levels. On the one hand, proteins and their hydrolysates could act as physical barriers, inhibiting the contact/binding of enzymes to starch^10^. On the other hand, the interactions between proteins/protein hydrolysates and starch improved the stability of starch structures and hindered the hydrolysis of digestive enzymes^11^. In addition, some studies^12,13^ have shown that proteins and their hydrolysates reduced starch digestion by inhibiting α-amylase activity. Many studies have explored factors that influence starch-protein interactions and complexation to modulate the properties of starch-protein complexes. For example, the source and amount of protein significantly altered the starch-protein interactions, which further changed the stability of starch structure and the rate and extent of in vitro digestion^9,12^.

Rice protein has been validated as a suitable protein source for the prevention of obesity and diabetes^14^. Rice peptides (RP) are defined as protein fragments that have positive effects on body function and health^15,16^. Many studies have found that protein hydrolysates were more effective in hindering starch digestion than intact proteins, and protein hydrolysates hydrolyzed by pepsin and pepsin-pancreatin (60 or 120 min) had different effects on starch digestion^7,12^, but the exact reason is unknown. Protein hydrolysates contain many peptide fragments with different molecular weights. Here, we hypothesized that peptide fragments of different molecular weights in protein hydrolysates played different roles in mitigating starch digestion. Therefore, in this study, peptides with different molecular weights in rice protein hydrolysates were obtained by ultrafiltration and their effects on the glucose release rate of gelatinized starch in vitro were explored. The effects of RP with different molecular weights on the digestibility of rice starch were investigated by determining the thermal properties, leached amylose amount of starch, starch structure (crystals and lamellae), and starch-peptide interactions. Data from the current work contribute to further our understanding of the starch-protein interactions and have implications for the preparation of carbohydrate-based foods with low starch digestibility.

## Results and discussion

### In vitro starch digestibility in the presence of RP

The in vitro digestion results for rice starch before and after the addition of RP are shown in Table 1. The rapidly digestible starch (RDS), slowly digestible starch (SDS), and resistant starch (RS) contents of rice starch were 68.02, 14.44, and 17.54 g/100 g, respectively. The digestion behavior of the starch-peptide mixtures was obviously mitigated compared to that of rice starch. The RDS content decreased to 45.90–63.84 g/100 g, and the RS content increased to 24.15–36.54 g/100 g. In addition, in the range of 0–10%, the content of RDS decreased and the content of RS increased with increased peptide addition. Previous studies^17^ have found that some grain peptides can bind to amylase and inhibit its activity, which may explain why the RDS content of 10%-RP-starch is lower than that of 5%-RP-starch. Compared to RP_1–5_ and RP_5–10_, 10%-RP_>10_ formed less RDS (45.90 g/100 g) and more RS (36.54 g/100 g). This phenomenon might result from the large steric hindrance of peptide fragments with large molecular weights, which forms a physical barrier to starch and prevents the combination of partial digestive enzymes and starch. This might also result from the large number of amino acids in the peptide fragment with large molecular weight, which formed a strong bond with starch during the process of co-gelatinization, thereby inhibiting its hydrolysis.

**Table 1:** The values for RDS, SDS, RS fractions, and digestion rate coefficient of RP-starch complexes.

Addition	Samples	RDS (g/100 g)	SDS (g/100 g)	RS (g/100 g)	*k* (10^−2^, min^−1^)
0%	Starch	68.02 ± 0.08^a^	14.44 ± 0.11^c^	17.54 ± 0.23^e^	8.71 ± 0.11^c^
5%	RP_1-5_-starch	63.84 ± 0.13^b^	12.01 ± 0.23^d^	24.15 ± 0.36^d^	9.22 ± 0.04^b^
	RP_5-10_-starch	52.97 ± 0.22^d^	14.59 ± 0.51^c^	32.44 ± 0.24^c^	7.66 ± 0.13^a^
	RP_>10_-starch	52.06 ± 0.04^e^	11.63 ± 0.26^d^	36.31 ± 0.05^a^	8.50 ± 0.05^d^
10%	RP_1-5_-starch	60.65 ± 0.18^c^	4.71 ± 0.75^e^	34.64 ± 0.17^b^	13.15 ± 0.09^a^
	RP_5-10_-starch	46.21 ± 0.15^f^	18.77 ± 0.28^a^	35.02 ± 0.13^b^	6.20 ± 0.12^f^
	RP_>10_-starch	45.90 ± 0.26^g^	17.56 ± 0.09^b^	36.54 ± 0.29^a^	6.42 ± 0.05^e^

Each value is the mean of three replicates. For the same column, data with same letters do not differ significantly from each other whereas data with different superscripts differ significantly at the probability level *p* < 0.05.

To further understand the mechanism of starch digestion, the data were fitted to a first-order kinetic equation to obtain the hydrolysis kinetic constant (*k*). The *k* value reflects the sensitivity of starch samples to amylase hydrolysis. As shown in Table 1, the kinetic constant of rice starch is 8.71 × 10^−2 ^min^−1^. Except for RP_1–5_, both RP_5–10_ and RP_>10_ significantly decreased the kinetic constant of starch (6.20 × 10^−2^~8.50 × 10^−2 ^min^−1^). These results show that both RP_5–10_ and RP_>10_ reduced the digestion rate of rice starch, which is consistent with the reduction in RDS content. The complexation of RP_1–5_ increased the kinetic constant of starch to 9.22 × 10^−2^ and 13.15 × 10^−2 ^min^−1^, presumably because the complexation of RP_1-5_ resulted in the conversion of part of SDS to RS. To verify this conjecture, subsequent studies explored the effects of RP affecting starch digestion in terms of thermal characteristics, leached amylose amount during gelatinization, and starch structure (crystal and lamellar).

### Effects of RP on the thermal properties of starch

The effect of RP with different molecular weights on the thermal properties of starch was determined using differential scanning calorimeter (DSC). The gelatinization temperatures (*T*_o_, *T*_p_, *T*_c_) and enthalpy changes (Δ*H*) were listed in Table 2. All blends had a distinct single endothermic peak, which was the result of the double helix dissociation of amylopectin molecules^18^. The *T*_o_, *T*_p_, and *T*_c_ values of rice starch were 64.47, 69.73, and 77.13 °C, respectively, and Δ*H* was 10.03 J/g. The gelatinization temperature and enthalpy of rice starch increased with the addition of peptides with different molecular weights. With an increase in peptide content from 0 to 10%, the gelatinization temperature of starch increased continuously, but the gelatinization enthalpy decreased.

**Table 2:** Thermal properties of RP-starch complexes.

Addition	Samples	T_O_/°C	T_P_/°C	T_C_/°C	ΔH/J·g^−1^
0%	Starch	64.47 ± 0.18^e^	69.73 ± 0.23^e^	77.13 ± 0.31^f^	10.03 ± 0.11^e^
5%	RP_1-5_-starch	64.72 ± 0.24^e^	72.37 ± 0.25^c^	79.44 ± 0.18^cd^	12.08 ± 0.17^cd^
	RP_5-10_-starch	67.15 ± 0.27^c^	72.44 ± 0.19^c^	79.77 ± 0.19^c^	12.44 ± 0.24^b^
	RP_>10_-starch	64.61 ± 0.08^e^	69.78 ± 0.29^e^	78.87 ± 0.23^e^	13.67 ± 0.26^a^
10%	RP_1-5_-starch	69.40 ± 0.05^a^	74.59 ± 0.06^a^	81.50 ± 0.07^a^	11.92 ± 0.08^d^
	RP_5-10_-starch	67.78 ± 0.13^b^	73.23 ± 0.02^b^	80.79 ± 0.28^b^	12.36 ± 0.03^bc^
	RP_>10_-starch	65.22 ± 0.18^d^	70.81 ± 0.31^d^	79.23 ± 0.30^de^	12.47 ± 0.13^b^

Each value is the mean of three replicates. For the same column, data with same letters do not differ significantly from each other whereas data with different superscripts differ significantly at the probability level *p* < 0.05.

Meanwhile, the addition of 5% peptide increased the onset gelatinization temperature of starch to 64.61–67.15 °C, and the addition of 10% peptide increased the onset gelatinization temperature of starch to 65.22–69.40 °C. The ΔH values also increased to 12.08–13.67 and 11.92–12.47 J/g, respectively. In addition, the complexes of RP with a molecular weight >10 kDa had the highest ΔH values, which were 13.67 J/g (5% addition) and 12.47 J/g (10% addition). The results show that, in the presence of RP_>10_, destruction of the starch structure required the greatest energy. This also explains why the RS content was the highest in the RP_>10_-starch complex. Therefore, the addition of RP delays the gelatinization of starch granules. This may be related to interactions between rice starch and peptides.

### Leached amylose amount

**Fig. 1:**
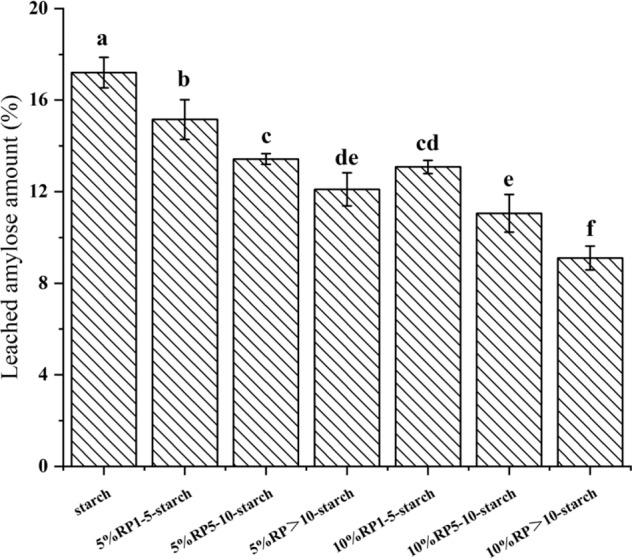
The leached amylose amounts of native starch and RP-starch mixtures. The error bars on the bar charts represented the standard deviations. Column diagrams with different letters indicated significant difference (*p* < 0.05).

During the gelatinization process, a large number of water molecules migrated into the starch granules, resulting in sufficient starch swelling and amylose leaching^19^. As shown in Fig. 1, the leached amylose content of the rice starch/peptide system was significantly lower than that of the gelatinized starch. The addition of RP with different molecular weights reduced the leached amylose content of rice starch from 17.2% to 9.1%–15.15%. This result is similar to previously reported changes in starch/protein systems^12^. The presence of rice proteolytic peptides prevents the diffusion of amylose during gelatinization. Within the range of 0–10% RP addition, the leached amylose amount of the starch/peptide system decreased with an increase in RP content. This may be because of the formation of complexes between amylose and peptide chains through intermolecular hydrogen bonds or hydrophobic interactions^20^, thereby improving the thermal stability of starch. In addition, the amount of leached amylose decreased in the order RP_>10_-starch < RP_5–10_-starch < RP_1–5_-starch. This is consistent with the thermal stability results (ΔH) obtained from the DSC analysis.

### Crystalline structure and long-range ordered structure

**Fig. 2:**
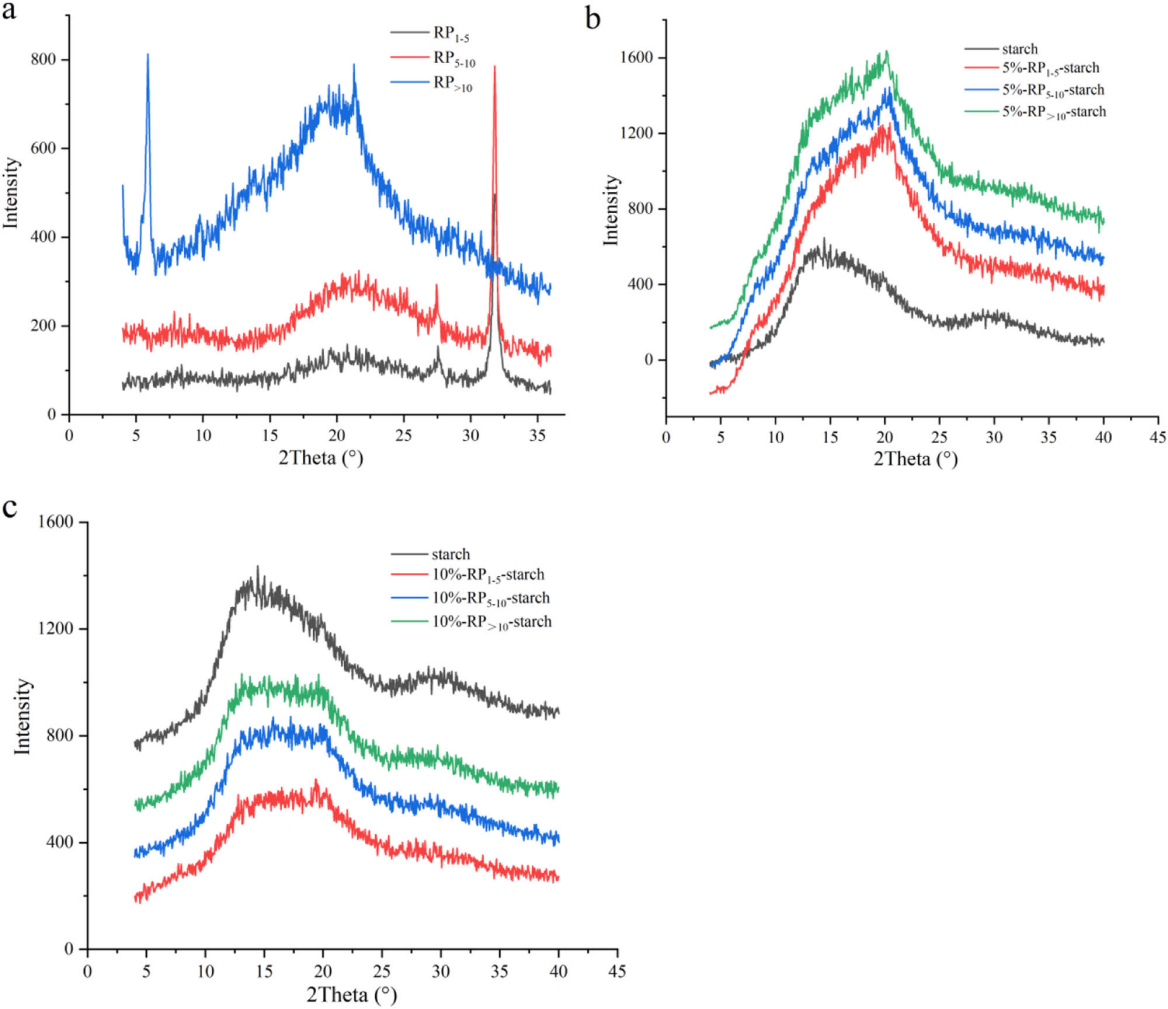
X-ray diffraction patterns of RP, 5% RP-starch complexes, and 10% RP-starch complexes. **a** X-ray diffraction patterns of rice peptides with different molecular weights (1–5, 5–10, and >10 kDa). **b** X-ray diffraction patterns of 5% RP-starch complexes. **c** X-ray diffraction patterns of 10% RP-starch complexes.

Native rice starch generally presents diffraction peaks at 15°, 17°, 18°, 20°, and 23° (2θ), showing a typical A + V-type crystal structure^21^. As shown in Fig. 2 and Table 3, the crystal structure of rice starch was almost completely destroyed after hydrothermal treatment, with diffraction peaks at 13° and 20° (2θ), and the relative crystallinity (RC) value was reduced to 3.91%. Complexation with RP significantly changed the crystal structure and RC value of starch. Compared with rice starch, the diffraction peak intensity at 20° (2θ) was higher, which might result from some hydrophobic peptides forming inclusion complexes with starch. The results indicate that the slowing of rice starch digestion in vitro was related to the V-type complexes formed by RP and starch to some extent. In addition, there was no significant difference in the intensities of the V-type peaks formed by RP with different molecular weights and starch. Compared to 10% RP, 5% RP formed more complexes with starch. We speculate that 10% RP have relatively strong electrostatic or hydrophobic interactions with starch, thereby reducing complex formation. Therefore, the RC value of the 10%-RP-S was lower than that of the 5%-RP-S.

**Table 3:** The XRD, FTIR spectra, and SAXS analysis parameters of RP-starch complexes.

Addition	Samples	RC/%	*R*/_995/1022_	*α*
0%	Starch	3.91 ± 0.31^f^	0.947 ± 0.008^d^	1.55 ± 0.02^e^
5%	RP_1–5_-starch	10.17 ± 0.14^b^	1.014 ± 0.003^c^	1.92 ± 0.03^b^
	RP_5–10_-starch	10.40 ± 0.15^b^	1.025 ± 0.007^b^	1.83 ± 0.02^c^
	RP_>10_-starch	12.46 ± 0.24^a^	1.034 ± 0.003^a^	1.97 ± 0.05^ab^
10%	RP_1–5_-starch	5.98 ± 0.17^e^	1.007 ± 0.001^c^	2.00 ± 0.02^a^
	RP_5–10_-starch	6.93 ± 0.14^d^	1.008 ± 0.005^c^	1.98 ± 0.05^ab^
	RP_>10_-starch	7.30 ± 0.08^c^	1.013 ± 0.003^c^	1.67 ± 0.06^d^

Each value is the mean of three replicates. For the same column, data with same letters do not differ significantly from each other whereas data with different superscripts differ significantly at the probability level *p* < 0.05.

The addition of different amounts of RP (0, 5, and 10%) and molecular weights (1–5, 5–10, and >10 kDa) increased the RC value of rice starch (5.98–12.46%), showing that RP significantly enhanced the long-range ordered degree of cooked starch. This result can be attributed to the interaction between the side chain groups of RP and starch chains during the co-gelatinization process, which promotes the rearrangement of starch chains. Moreover, with an increase in the molecular weight of RP, the RC value of RP-starch increased gradually. These results indicate that the relatively strong interaction between RP with larger molecular weights (RP_>10_) and starch chains promoted the ordered degree of cooked starch and further enhanced the ability of starch granules to resist enzymatic hydrolysis.

### Chemical structure and short-range ordered structure

**Fig. 3:**
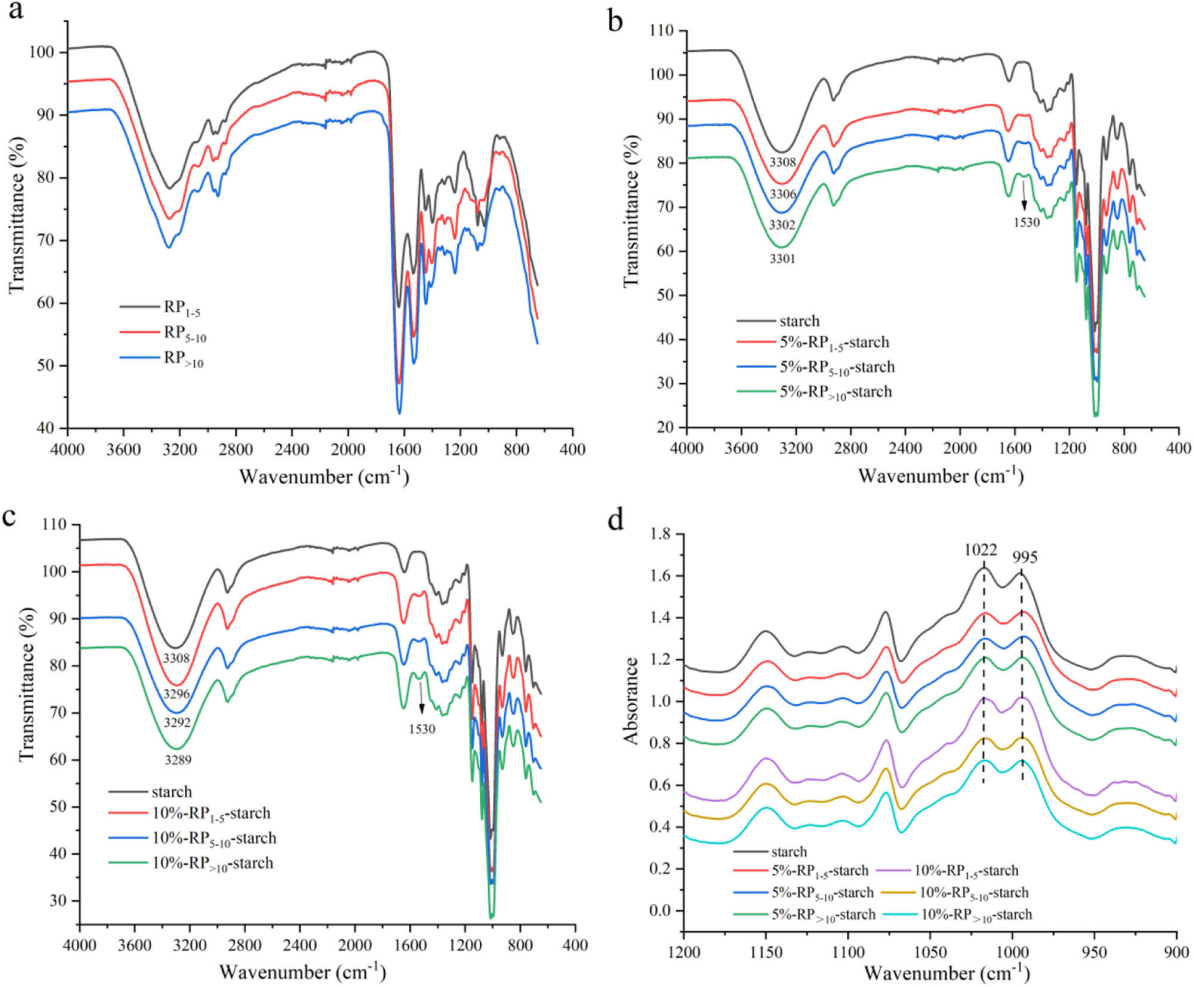
FTIR and deconvoluted FTIR spectra of cooked starch and RP-starch complexes. **a** FTIR spectra of rice peptides with different molecular weights (1–5, 5–10, and >10 kDa). **b** FTIR spectra of 5% RP-starch complexes. **c** FTIR spectra of 10% RP-starch complexes. **d** Deconvoluted FTIR spectra of 5% RP-starch complexes and 10% RP-starch complexes.

The Fourier transform infrared spectroscopy (FTIR) spectra of the cooked starch and RP-starch mixtures are shown in Fig. 3. Compared with the starch after hydrothermal treatment, the shape of the infrared absorption peak of RP-starch did not change significantly, and no new groups appeared in the system, indicating that there were no covalent interactions between rice starch and RP^22^. The band at 2930 cm^−1^ is related to the C–H stretching vibration. The band at 1530 cm^−1^ of the RP-starch mixtures is related to the deformation absorption peak of N-H^23^, and the peak intensity gradually increased with increasing RP addition amount. The absorption peaks at 1168, 1084, and 1014 cm^−1^ were attributed to C–O bond stretching, and the addition of RP reduced the absorption intensity of C–O. The bands in the range of 3000–3600 cm^−1^ are related to the O–H stretching vibration of starch molecules or the N-H stretching vibration of protein molecules^24^. The shift of the peak position to lower wavenumbers indicates the formation of hydrogen bonds in the system^7^. The addition of RP shifted the absorption peak at 3308 cm^−1^ to 3289–3306 cm^−1^, indicating that there was hydrogen bonding between RP and starch, which verified our speculation. In addition, in the range of 0–10%, the higher the amount of RP added, the more hydrogen bonds were formed with starch. Moreover, the order of hydrogen bonding with starch was as follows: RP_>10_ > RP_5–10_ > RP_1–5_. These results show that the higher the molecular weight, the more hydrogen bonds formed between the RP groups and the hydroxyl groups in the starch and the stronger the interaction, which is consistent with the XRD analysis results. In conclusion, the hydrogen bonding between RP and starch during co-gelatinization is related to the amount of RP added and the molecular weight. The hydrogen bond between RP-starch inhibited the interaction between starch molecular chains and played an important role in the RP-mediated inhibition of starch digestion.

Deconvoluted FTIR spectra of 1200–900 cm^−1^ were used to analyze the effects of RP addition on the short-range ordered structure of rice starch. The bands at 1022 and 995 cm^−1^ are related to the amorphous material and short-range order of starch. Usually, the ratio of the absorbance values at 995 and 1022 cm^−1^ (*R*_995/1022_) is used to characterize the change in the molecular order of starch^25^. As shown in Table 3, the *R*_995/1022_ value of cooked starch was 0.947, and the addition of RP increased the *R*_995/1022_ value of starch to 1.007–1.034. The results indicate that the presence of RP promoted the short-range arrangement of starch chains during thermal processing and improved the short-range-ordered structure. When the addition of RP increased from 5 to 10%, the *R*_995/1022_ value of the RP-starch mixtures showed an apparent decrease in *R*_995/1022_. In other words, the presence of excessive RP inhibited the rearrangement of starch chains during thermal processing. In addition, mixtures of starch and RP with relatively large molecular weights displayed a slight increase in *R*_995/1022_. More specifically, RP with large molecular weights were more likely to interact with starch than peptides with small molecular weights; thus, rice starch formed a more ordered short-range structure during the gelatinization process.

### Lamellar structure and fractal characteristics

**Fig. 4:**
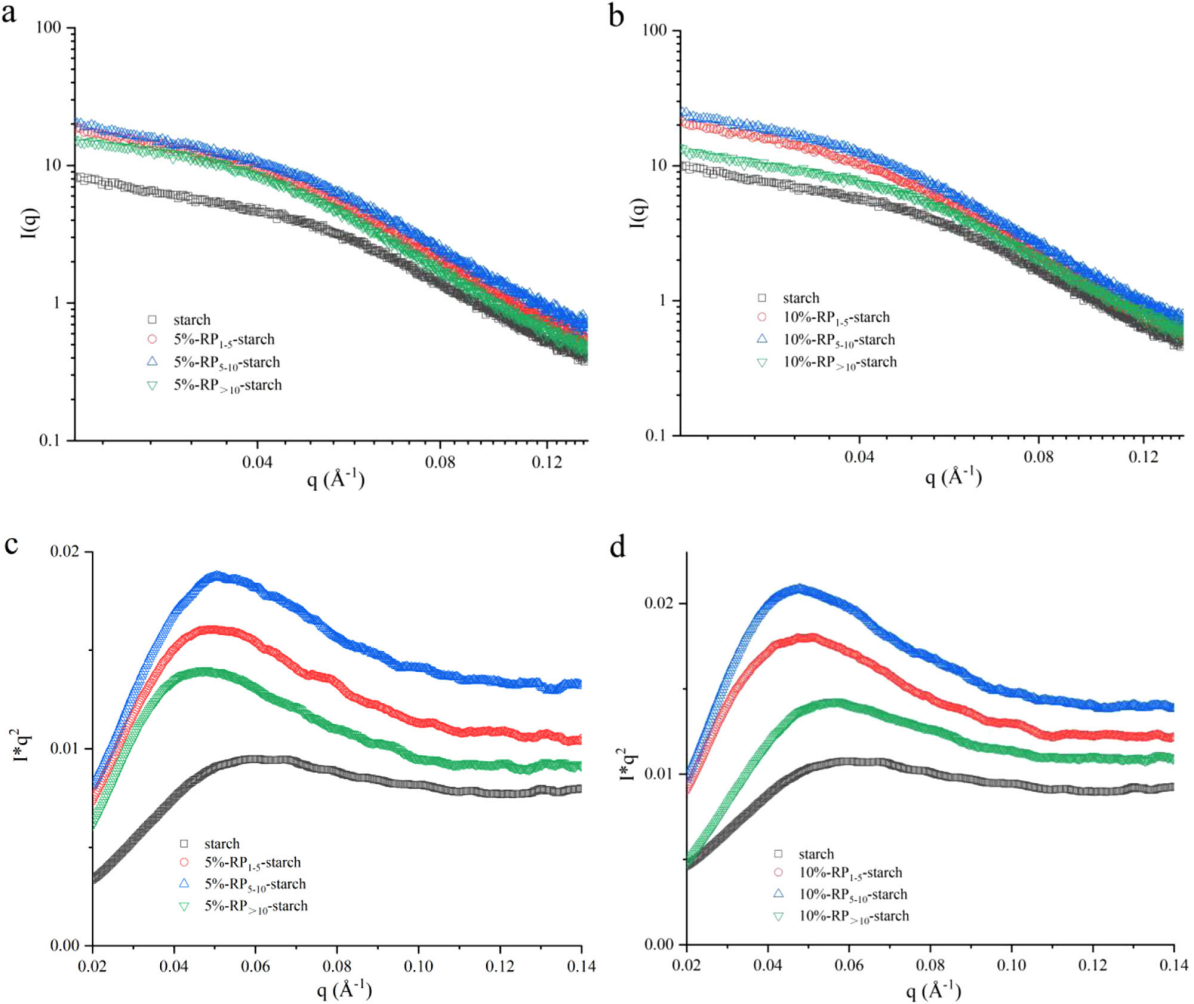
SAXS curves and Kratky plots of cooked starch and RP-starch complexes. **a** SAXS curves of 5% RP-starch complexes. **b** SAXS curves of 10% RP-starch complexes. **c** Kratky plots of 5% RP-starch complexes. **d** Kratky plots of 10% RP-starch complexes.

The aggregate structures of the rice starch-rice peptide complexes were further analyzed by small-angle X-ray scattering (SAXS). As shown in Fig. 4, the SAXS curves of all starch samples showed a shoulder-like peak around 0.05 Å^−1^, but the scattering intensities on the SAXS curves of different complexes were different. Owing to the different structures of peptides with different molecular weights, the interactions between them and starch were also different, which induced the formation of different aggregated structures^12^. In the SAXS tests, the scattering peak intensity was determined by the number of well-organized semicrystalline structures and/or the difference in electron density between the crystalline and amorphous lamellae in the starch. The larger the electron density, the higher the intensity of the scattering peak on the SAXS curve^26^. Therefore, it can be seen that the presence of RP significantly enhanced the scattering peak intensity of rice starch, which resulted from the rearrangement of starch chains in the presence of peptides during hydrothermal treatment and enhanced the electron density difference between the two types of lamellae^27^. Combined with the XRD analysis, the increase in electron density is associated with an increase in the ordered structure. Meanwhile, the complexation with RP shifted the scattering peak of the starch paste to a lower *q* position (closer to 0.04 Å^−1^), showing that the addition of RP increased the size of the aperiodic structure of starch paste. The complex formed by starch and 5% RP_>10_ had the largest aperiodic structure.

Furthermore, a Lorentz correction was performed to make the scattering peaks more distinct using a Kratky plot (*q*^2^*I*(*q*) vs. *q*). As shown in Fig. 4c, d, a characteristic peak was observed in the range of 0.02 < *q* < 0.1 Å^−1^, indicating that the starch-peptide complexes had certain aggregation structures^28^. The intensities of the peaks had a similar trend to that shown in Fig. 4a, b, and the addition of RP significantly increased the peak area of the rice starch paste. This result further indicates that the complexation of starch with RP increased the density of the starch paste, and the degree of increase depended on the molecular weight and addition amount of the peptides. Combined with the in vitro digestion analysis, the increase in structural compactness largely hindered the enzymatic hydrolysis of starch granules, thereby reducing the rate and extent of starch hydrolysis.

To investigate the surface smoothness and compactness of the rice starch-peptide structure, the D characteristics of the surface/mass fractal structure were further analyzed according to the scattering power-law equation (*I*–*q*^−α^). As shown in Table 3, the α value of rice starch was 1.55, and the α value increased to 1.67–2.00 after the addition of RP, all of which showed a mass fractal structure^29^. This is because the migration of water molecules breaks the hydrogen bonds between starch chains and induces the recombination of starch and peptide chains to form new aggregates during the gelatinization process, thus forming a higher-density aggregate structure. In addition, the complexes formed by RP_>10_ with starch were more compact than those formed by RP_1–5_ and RP_5–10_ when the peptide content was 5%. However, when the peptide content was 10%, compared to RP_1–5_ and RP_5–10_, RP_>10_ and rice starch formed a relatively loose aggregate structure. This result also indicates that the structural compactness of the aggregates formed by the peptide and starch was related to the molecular weight and addition amount of RP. In conclusion, the scatterers of RP-starch complexes were denser, and the surface smoothness was higher than that of rice starch. Combined with FTIR analysis, the results suggest that the changes in the fractal structure were related to the hydrogen bond network formed between the starch chains and RP.

### Mechanism of RP-mediated inhibition of starch digestion

Rice starch is the most important starch-based food in the daily diet and is mainly consumed with other components such as lipids and proteins. Rice protein has been validated as a suitable protein source for the prevention of obesity and diabetes^10,30^. RP are defined as protein fragments that have positive effects on body function and health^31^. In this study, we show that RP with different molecular weights in the protein hydrolysates effectively slowed down starch digestion in starch matrices. In the presence of RP, especially RP_>10_, rice starch showed higher thermal stability and more energy was required to break its structure (Table 2). During starch gelatinization, the hydrogen bonding interaction between the starch and peptide chains induced the rearrangement of starch chains and improved its structural order (Fig. 3). RP_>10_ formed more hydrogen bonds with starch. In addition, rice starch-RP had a denser agglomerate structure with less leached amylose (Figs. 1 and 4). The presence of some hydrophobic peptides also slightly increased the V-type structure of starch (Fig. 2), which hindered its hydrolysis by digestive enzymes.

## Conclusion

This study investigated the effects of RP of different molecular weights on the structure, thermal properties, and digestive properties of rice starch. All added peptides mitigated the digestion of rice starch in the range of 0–10%. RP, particularly RP_>10_, improved the thermal stability of starch under hydrothermal conditions and increased the compactness of the aggregated structure. In addition, the hydrogen bond interactions between the peptide chains and starch chains played an important role in hindering starch digestion. The presence of hydrophobic peptides also slightly increased V-type peak intensity. These findings enrich our understanding of starch-peptide interactions and have important implications for the development of carbohydrate-restricted diets and protein functional foods.

## Methods

### Materials

Rice starch and protein were obtained from Jiangsu Jinnong (Jiangsu, China). Pepsin (≥250 U/mg, P7000, EC 3.4.23.1), pancreatin from porcine pancreas (8 × USP, P7545, EC 232-468-9), and glucoamylase (260 U/mL, A7095, EC 3.2.1.3) were purchased from Sigma-Aldrich (St. Louis, MO, USA). Glucose oxidase-peroxidase assay kits (GOPOD) were obtained from Leadman Biochemistry (Beijing, China). All other chemicals and reagents used were of analytical grade.

### Preparation of samples

#### Preparation of RP with different molecular weights

Rice protein (10 g) was weighed and dissolved in 200 mL of hydrochloric acid water (pH = 1.2) containing pepsin from the porcine gastric mucosa (2000 U/mL). After enzymatic hydrolysis for 120 min, the pH was adjusted to 7 with sodium hydroxide solution (1 mol/L) to inactivate the pepsin. The above enzymatic reactions were all carried out in a water bath at 37 °C with shaking at 150 rpm. The rice protein hydrolysates were then separated and fractionated by polysulfone ultrafiltration membrane with molecular cutoff values of 10, 5, and 1 kDa, successively, to obtain RP with molecular weights of 0–10, 5–10, and 1–5 kDa.

#### Preparation of rice starch-rice peptide mixtures

Rice starch (5 g, dry basis) was accurately weighed and 50 mL of distilled water was added to prepare the starch suspension. Starch-based peptides (0, 5, and 10%) with different molecular weights (1–5, 5–10, and >10 kDa) were added to the starch suspension. Then the mixtures were incubated in a water bath at 100 °C and a shear rate of 400 rpm for 30 min. Subsequently, the starch paste was cooled to 25 °C and then freeze-dried for further analyses. The blank group consisted of gelatinized starch without adding peptides. The mixtures were labeled as 0%, 5%, 10% RP_1–5_-starch, RP_5–10_-starch, and RP_>10_-starch.

#### In vitro digestion of starch samples

According to Lu et al.^7^, 4.5 g of pancreatin was evenly dispersed in deionized water (40 mL) and centrifuged at 5000 × *g* for 10 min. Then, 27 mL of the supernatant was mixed with 3.2 mL of glucoamylase to prepare the enzyme mixtures. Starch sample (~200 mg) was stirred with deionized water (2 mL). Pepsin (20 mg) was added to 4 mL HCl (0.05 mol/L) and stirred evenly. Then the pepsin solution was added into the sample centrifuge tube and shook for 30 min. After pepsin hydrolysis, 10 glass beads (3–4 mm in diameter) and 2 mL sodium acetate buffer (0.5 mol/L, pH 5.2) were added to the tube. The samples were placed in a water bath (37 °C, 170 rpm) for 30 min, and the enzyme mixtures (2 mL) were added to the starch samples. At some time interval (0, 10, 20, 30, 45, 60, 90, 120, 150, and 180 min), the enzymatic hydrolysates (0.1 mL) were removed and placed in 0.9 mL of ethanol solution (90%) to terminate the enzymatic hydrolysis reaction. The glucose content of the starch samples was determined using the GOPOD method. The RDS, SDS, and RS contents were calculated using the following formulas:

1 $${\rm{RDS}}\left( {g/100g} \right) = G20 \ast 0.9/Ws \ast 100$$

2 $${\rm{SDS}}\left( {g/100g} \right) = \left( {G120 - G20} \right) \ast 0.9/Ws \ast 100$$

3 $${\rm{RS}}\left( {g/100g} \right) = 100 - {\rm{RDS}} - {\rm{SDS}}$$

G20: glucose released after 20 min; G120: glucose released after 120 min; Ws: weight of the sample.

The in vitro digestibility curve of the starch samples was fitted to the following first-order equation:

4 $$C_t = C_\infty \left( {1 - e^{ - kt}} \right)$$

where *C*_∞_ refers to the glucose concentration at the end of the reaction, *C*_t_ refers to the glucose concentration at time *t*, and *k* (min^−1^) is the reaction rate constant of the starch samples.

#### Determination of thermal properties

The effects of RP with different molecular weights on the thermal properties of rice starch were investigated using differential scanning calorimetry^32^. Native starch and starch-peptide mixtures (4.0 mg) and distilled water (8 µL) were added to DSC crucibles. The crucibles were equilibrated at 25 °C overnight. Each sample was heated from 20 to 120 °C at a rate of 10 °C/min. An empty crucible served as the reference.

#### Determination of leached amylose amount

The iodine colorimetric method was used to determine the amount of amylose leached from the starch-peptide mixed system during gelatinization. The native starch or starch-peptide mixed system was incubated at 95 °C for 30 min and then kept at 37 °C for another 20 min. Subsequently, starch paste (1.0 g) was completely dispersed in 5 mL of distilled water and centrifuged for 20 min (5000 × *g*). The supernatant (0.5 mL) was then mixed with a sodium hydroxide solution (3 mL, 0.1 mol/L). After incubation for 10 min in boiling water and rapid cooling, acetic acid (0.3 mL, 1 mol/L) and I_2_-KI aqueous solution (0.5 mL, 0.1 mol/L) were added. After incubating in the dark for 10 min (25°C), the absorbance values were measured at 620 nm using a UV-Vis spectrophotometer (TU-1900, Beijing General Instrument Co. Ltd.). Then the leached amylose amount was calculated by dividing the amylose content in the supernatant by the original weight of rice starch^33^.

#### XRD analysis

Under the conditions of 44 kV and 30 mA, XRD with a Cu-Kα radiation source (λ = 0.1542 nm) was used to determine the effects of RP with different molecular weights on the crystalline and long-ordered structures of rice starch. The samples were scanned from 4 to 40° (2θ) at a scan rate of 5°/min. The relative crystallinity (RC) of the samples was calculated using the MDI Jade 6.0.

5 $$RC\left( {{{\mathrm{\% }}}} \right) = \frac{{A_c}}{{A_c + A_a}} \times 100\%$$

Here *A*_c_ refered to the area of the crystalline peak and *A*_a_ refered to the area of the amorphous peak.

#### Attenuated total reflectance-Fourier transform infrared spectroscopy (ATR-FTIR) analysis

An ATR-FTIR spectrometer was used to determine the effects of RP with different molecular weights on the short-ordered structure of rice starch. An appropriate amount of the powder sample was taken and evenly distributed in the middle of the ATR accessory sample table. The acquisition range was 4000–400 cm^−1^. The FTIR spectra were processed using OMNIC 8.0 through automatic baseline correction, automatic smoothing, and Fourier deconvolution. The deconvolution half-band width was 21 cm^−1^ and the enhancement factor was 2.1.

#### SAXS analysis

The starch samples were pretreated according to a previously described method^26^. Starch was suspended in deionized water at a mass ratio of 1:3 and shaken overnight at 25 °C to reach equilibrium. Before the experiment, the starch slurry was centrifuged for 5 min (5000 × *g*). The scattering patterns were then obtained using a Xeuss 3.0 C SAXS instrument. The wavelength (λ) was 1.542 nm, and the sample-detector distance was 1069 mm. The exposure time was 60 s and the q range was 0.01–0.2 Å^−1^.

To further investigate the lamellar structure of the starch samples, SAXS curves were transformed using the one-dimensional linear correlation function, γ(x):

6 $${\upgamma}\left( x \right) = \frac{{{\int}_0^\infty {I\left( q \right)q^2\cos \left( {qx} \right)dq} }}{{{\int}_0^\infty {I\left( q \right)q^2dq} }}$$

where X refers to the distance in real space and ${\int}_0^\infty {I\left( q \right)q^2} dq$ refers to the scattering invariant.

### Statistical analysis

The data were presented as mean ± standard deviation, with at least three replicates per experiment. Statistical significance of data was determined with Duncan’s test (*p* < 0.05) and assessed with SPSS Statistics 20.0 software (IBM SPSS, Armonk, NY, USA).

## Data Availability

The datasets generated during and/or analyzed during the current study are available from the corresponding author on reasonable request.

## References

[1] Sen S, Chakraborty R, Kalita P (2020). Rice-not just a staple food: a comprehensive review on its phytochemicals and therapeutic potential. Trends Food Sci. Technol..

[2] Mitra A, Bhattacharya D, Roy S (2007). Role of resistant starches particularly rice containing resistant starches in type 2 diabetes. J. Hum. Ecol..

[3] Wong THT, Louie JCY (2017). The relationship between resistant starch and glycemic control: a review on current evidence and possible mechanisms. Starch‐Stärke.

[4] Dreher ML, Dreher CJ, Berry JW, Fleming SE (1984). Starch digestibility of foods: a nutritional perspective. Crit. Rev. Food Sci. Nutr..

[5] Singh J, Dartois A, Kaur L (2010). Starch digestibility in food matrix: a review. Trends Food Sci. Technol..

[6] Jung EY (2010). Feeding silk protein hydrolysates to C57BL/KsJ-db/db mice improves blood glucose and lipid profiles. Nutr. Res..

[7] Lu X, Ma R, Qiu H, Sun C, Tian Y (2021). Mechanism of effect of endogenous/exogenous rice protein and its hydrolysates on rice starch digestibility. Int. J. Biol. Macromol..

[8] Manders RJ (2006). Co-ingestion of a protein hydrolysate with or without additional leucine effectively reduces postprandial blood glucose excursions in type 2 diabetic men. J. Nutr..

[9] Yang C, Zhong F, Goff D, Li Y (2019). Study on starch-protein interactions and their effects on physicochemical and digestible properties of the blends. Food Chem..

[10] López-Barón N, Gu Y, Vasanthan T, Hoover R (2017). Plant proteins mitigate in vitro wheat starch digestibility. Food Hydrocoll..

[11] Li C (2021). Effects of endogenous proteins on rice digestion during small intestine (in vitro) digestion. Food Chem..

[12] Chi C, Li X, Zhang Y, Chen L, Li L (2018). Understanding the mechanism of starch digestion mitigation by rice protein and its enzymatic hydrolysates. Food Hydrocoll..

[13] Xu H, Zhou J, Yu J, Wang S, Wang S (2021). Mechanisms underlying the effect of gluten and its hydrolysates on in vitro enzymatic digestibility of wheat starch. Food Hydrocoll..

[14] Higuchi Y (2019). Rice endosperm protein administration to juvenile mice regulates gut microbiota and suppresses the development of high-fat diet-induced obesity and related disorders in adulthood. Nutrients.

[15] Chalamaiah M, Ulug SK, Hong H, Wu J (2019). Regulatory requirements of bioactive peptides (protein hydrolysates) from food proteins. J. Funct. Foods.

[16] Zaky AA, Abd El-Aty A, Ma A, Jia Y (2020). An overview on antioxidant peptides from rice bran proteins: extraction, identification, and applications. Crit. Rev. Food Sci. Nutr..

[17] Uraipong C, Zhao J (2018). In vitro digestion of rice bran proteins produces peptides with potent inhibitory effects on alpha-glucosidase and angiotensin I converting enzyme. J. Sci. Food Agr..

[18] Ding Y (2021). Effects of endogenous proteins and lipids on structural, thermal, rheological, and pasting properties and digestibility of adlay seed (*Coix lacryma-jobi* L.) starch. Food Hydrocoll..

[19] Tsutsui K, Katsuta K, Matoba T, Takemasa M, Nishinari K (2005). Effect of annealing temperature on glatinization of rice starch suspension as studied by rheological and thermal measurements. J. Agric. Food Chem..

[20] Chen X (2019). Structural, physicochemical, and digestibility properties of starch-soybean peptide complex subjected to heat moisture treatment. Food Chem..

[21] Witek M (2010). The structural and hydration properties of heat-treated rice studied at multiple length scales. Food Chem..

[22] Blk A, Mk B, Bsg B (2020). Impact of ultrasonication on functional and structural properties of Indian wheat (*Triticum aestivum* L.) cultivar starches. Int. J. Biol. Macromol..

[23] Muscat D (2014). Effect of spatial distribution of wax and PEG-isocyanate on the morphology and hydrophobicity of starch films. Carbohydr. Polym..

[24] Huang TT, Zhou DN, Jin ZY, Xu XM, Chen HQ (2016). Effect of repeated heat-moisture treatments on digestibility, physicochemical and structural properties of sweet potato starch. Food Hydrocoll..

[25] Warren FJ, Gidley MJ, Flanagan BM (2016). Infrared spectroscopy as a tool to characterise starch ordered structure-a joint FTIR-ATR, NMR, XRD and DSC study. Carbohydr. Polym..

[26] Zhai Y, Li X, Bai Y, Jin Z, Svensson B (2022). Maltogenic α-amylase hydrolysis of wheat starch granules: mechanism and relation to starch retrogradation. Food Hydrocoll..

[27] Boonna S, Tongta S (2018). Structural transformation of crystallized debranched cassava starch during dual hydrothermal treatment in relation to enzyme digestibility. Carbohydr. Polym..

[28] Zhen Y (2022). Increasing the pH value during thermal processing suppresses the starch digestion of the resulting starch-protein-lipid complexes. Carbohydr. Polym..

[29] He H (2020). Improving the in vitro digestibility of rice starch by thermomechanically assisted complexation with guar gum. Food Hydrocoll..

[30] Jenkins DJ (1987). The effect of starch-protein interaction in wheat on the glycemic response and rate of in vitro digestion. Am. J. Clin. Nutr..

[31] Udenigwe CC (2014). Bioinformatics approaches, prospects and challenges of food bioactive peptide research. Trends Food Sci. Technol..

[32] Zhang J, Zhang ML, Bai X, Zhang YK, Wang C (2022). The impact of high hydrostatic pressure treatment time on the structure, gelatinization and thermal properties and in vitro digestibility of oat starch. Grain Oil Sci. Technol..

[33] Chen L, Tong QY, Ren F, Zhu JL (2014). Pasting and rheological properties of rice starch as affected by pullulan. Int. J. Biol. Macromol..

